# Contrast-Enhanced Ultrasound in the Differential Diagnosis of Primary Thyroid Lymphoma and Nodular Hashimoto’s Thyroiditis in a Background of Heterogeneous Parenchyma

**DOI:** 10.3389/fonc.2020.597975

**Published:** 2021-01-07

**Authors:** Lulu Yang, Haina Zhao, Yushuang He, Xianglan Zhu, Can Yue, Yan Luo, Buyun Ma

**Affiliations:** ^1^ Department of Ultrasound, West China Hospital of Sichuan University, Chengdu, China; ^2^ Department of Pathology, West China Hospital of Sichuan University, Chengdu, China

**Keywords:** contrast-enhanced ultrasound, ultrasonography, Hashimoto’s thyroiditis, lymphoma, thyroid

## Abstract

**Objective:**

To investigate the diagnostic performance of contrast-enhanced ultrasound (CEUS) in the differentiation of primary thyroid lymphoma (PTL) and nodular Hashimoto’s thyroiditis (NHT) in patients with background of heterogeneous diffuse Hashimoto’s thyroiditis (HT).

**Methods:**

Sixty HT patients with 64 thyroid nodules (31 PTL and 33 NHT) who had undergone CEUS examination were included in this study. With histopathological results as the reference, we evaluated the imaging features of each nodule on both conventional ultrasonography (US) and CEUS. Quantitative CEUS parameters including peak intensity (PI), time to peak (TTP), and area under the time–intensity curve (AUC) were gathered in the nodule and background parenchyma. The ratio indexes of theses parameters were calculated by the ratio of the lesion and the corresponding thyroid parenchyma. Logistic regression and receiver operating characteristic (ROC) curves analyses of valuable US indicators were further preformed to evaluate the diagnostic capability of CEUS in discrimination of PTL and NHT.

**Results:**

Among all the observed US imaging features and CEUS parameters, 10 indicators showed significant differences between PTL and NHT (all P < 0.05). All the significant indicators were ranked according to the odds ratios (ORs). Eight of them were CEUS associated including imaging features of enhancement pattern, degree, homogeneity, and quantification parameters of PI, AUC, ratios of PI, AUC, and TTP, while indicators on conventional US, including vascularity and size ranked the last two with ORs less than 3. The five single CEUS parameters showed good diagnostic performance in diagnosis of PTL with areas under ROC curves of 0.72–0.83 and accuracies of 70.3–75.0%. The combination of CEUS imaging features and the ratios of PI, AUC, and TTP demonstrated excellent diagnostic efficiency and achieved area under ROC curve of 0.92, which was significantly higher than any of the five single parameters (all P < 0.05), with a sensitivity of 83.9%, specificity of 87.9%, and accuracy of 85.9%.

**Conclusions:**

CEUS is an efficient diagnostic tool in the differential diagnosis of PTL and NHT for patients with diffuse HT. Conjoint analysis of CEUS imaging features and quantification parameters could improve the diagnostic values.

## Introduction

Hashimoto’s thyroiditis (HT) is a common autoimmune thyroid disease and affects at least 2% of all females (1). It is characterized by the presence of serum anti-thryoglobulin (TgAb) and anti-peroxidase (TPOAb) antibodies, sometimes accompanied with hypothyroidism. Patients with HT are demonstrated at 40–80 times greater risk for developing primary thyroid lymphoma (PTL) compared to those without thyroiditis (2, 3). It has also been reported that approximately 80% of PTL patients have a history of HT (4, 5). Although PTL is an uncommon cause of malignancy, which accounts for 5% of all thyroid malignances and occurs in less than 3% of non-Hodgkin’s extra-nodal lymphoma (6, 7), it deserves more attention because therapeutic strategies of PTL is different from other kinds of thyroid neoplasm. Early diagnosis and appropriate treatment could avoid extensive surgery and improve prognosis (8, 9).

Typical clinical feature of PTL is the rapid enlargement of goiter with associated compressive symptoms. However, with the improvement of ultrasonography (US) resolution and application of US in thyroid nodular screening, many lymphomas could be detected at early phase before the start of rapid growth (10–12). Sonographic features such as marked hypoechogenicity, posterior acoustic enhancement, and hypervascularity may suggest PTL by conventional US (10, 13–15). These features are somewhat subjective, and the HT background with a heterogeneously decreased parenchymal echogenicity and increased vascularity may cause the overlap in US appearance between benign and malignant lesions, which further makes the diagnosis more challenging. Contrast-enhanced ultrasound (CEUS) is a relatively new technique using contrast agent to dynamically evaluate the micro-vascularity of lesions and parenchymal perfusion. Recently, some published articles have shown that CEUS is a promising diagnostic tool to improve the diagnostic accuracy of identifying both malignant and benign nodules (16–20). Nevertheless, the majority of the reported studies focused the malignancy on papillary thyroid carcinoma (PTC) and none of them concerned the capability of CEUS to predict PTL coexistence with HT.

On the other hand, among patients with HT, nodular Hashimoto’s thyroiditis (NHT), the focal form of HT, was observed in 20–60% of all thyroid nodules (21, 22). Compared with other benign thyroid nodules, NHT has been more illustrated as hypoechoic with ill-defined margins, and seems to share more malignant sonographic features with lymphoma (22–25). Therefore, we aimed to assess the diagnostic performance of CEUS imaging and the corresponding quantitative parameters in the differentiation of PTL and NHT in a background of heterogeneous diffuse HT.

## Materials and Methods

### Patients

This retrospective study was approved by the Ethics Committee of West China Hospital. Written informed consent was obtained from all patients before CUES examination. The records of patients between January 2016 and December 2019 who had undergone both conventional US and CEUS of thyroid at our hospital were reviewed. Among them, 72 patients who were clinically diagnosed with HT and whose nodules were histopathologically confirmed as HT or lymphoma were enrolled in this study. The exclusion criteria included: patients whose biopsy were not performed within one month after US examination (n=8); patients whose nodules were too large without enough perinodular parenchyma for CEUS quantitative analysis (n=4). Ultimately, 64 thyroid nodules in 60 patients were included in this study. The laboratory test including serum thyroid hormones (thyroid-stimulating hormone TSH, free T4, and free T3), thyroid peroxidase antibody (A-TPO), and thyroglobulin antibody (A-TG) were collected before biopsy. Thyroid hormone level was defined as normal when TSH, T3, and T4 were all within normal limits.

### Examination Protocol

All the US examinations were performed using an iU22 US system (Phillips Healthcare, Bothell, USA) equipped with a 5–12 MHz linear transducer for conventional US and a 3–9 MHz linear transducer for CEUS. Examinations were conducted by two radiologists with more than 9 years of experience in thyroid US scanning and at least 3 years of experience in CEUS performance.

Patients were instructed to lie in a supine position with neck hyperextended. All target nodules were evaluated on conventional B-mode US and color Doppler US. CEUS was performed using reverse pulse imaging technique with a real-time, low-mechanical index (MI = 0.06). In total, a bonus of 2.0 ml ultrasound contrast agent SonoVue (Bracco, Milan, Italy) was injected intravenously *via* a 20-gauge intravenous cannula, followed by a flush with 5 ml of saline solution (0.9% sodium chloride). The timer on US system was initiated simultaneously, and the CEUS dynamic videos were recorded continuously at least 90 s for further analysis.

### Imaging Interpretation

Two other radiologists with more than 10 years of experience in thyroid US diagnosis, who were blinded to the pathological results, independently evaluated and analyzed the conventional US and CUES images. Different opinions were discussed in consensus.

The conventional US features of each nodule were evaluated as follows: size on US (maximum diameter); number of suspicious nodules (solitary or multiple); shape (taller than wide or wider than tall); margin (well-defined or ill-defined); inner echogenicity (hypo-, iso-, or hyper-echoic); posterior acoustic enhancement (present or absent); echoic strands (present or absent); calcification (present or absent); vascular distribution (none, mainly internal, mainly peripheral or internal and peripheral mixed).

The CEUS imaging features including the enhancement pattern, degree, and homogeneity were assessed. According to the way contrast agent entered the nodules, the enhancement pattern was defined as centripetal perfusion (contrast agent fill the lesions from the peripheral region to the center) and synchronously perfusion (contrast agent synchronously fill the peripheral region and center of the lesions). With respect to the surrounding thyroid parenchyma, the enhancement degree of the lesion was classified as hypo-enhancement and iso- or hyper-enhancement. Based on whether the contrast agent was evenly distributed in lesions, the enhancement homogeneity was divided into homogeneous and heterogeneous.

**Figure 1:**
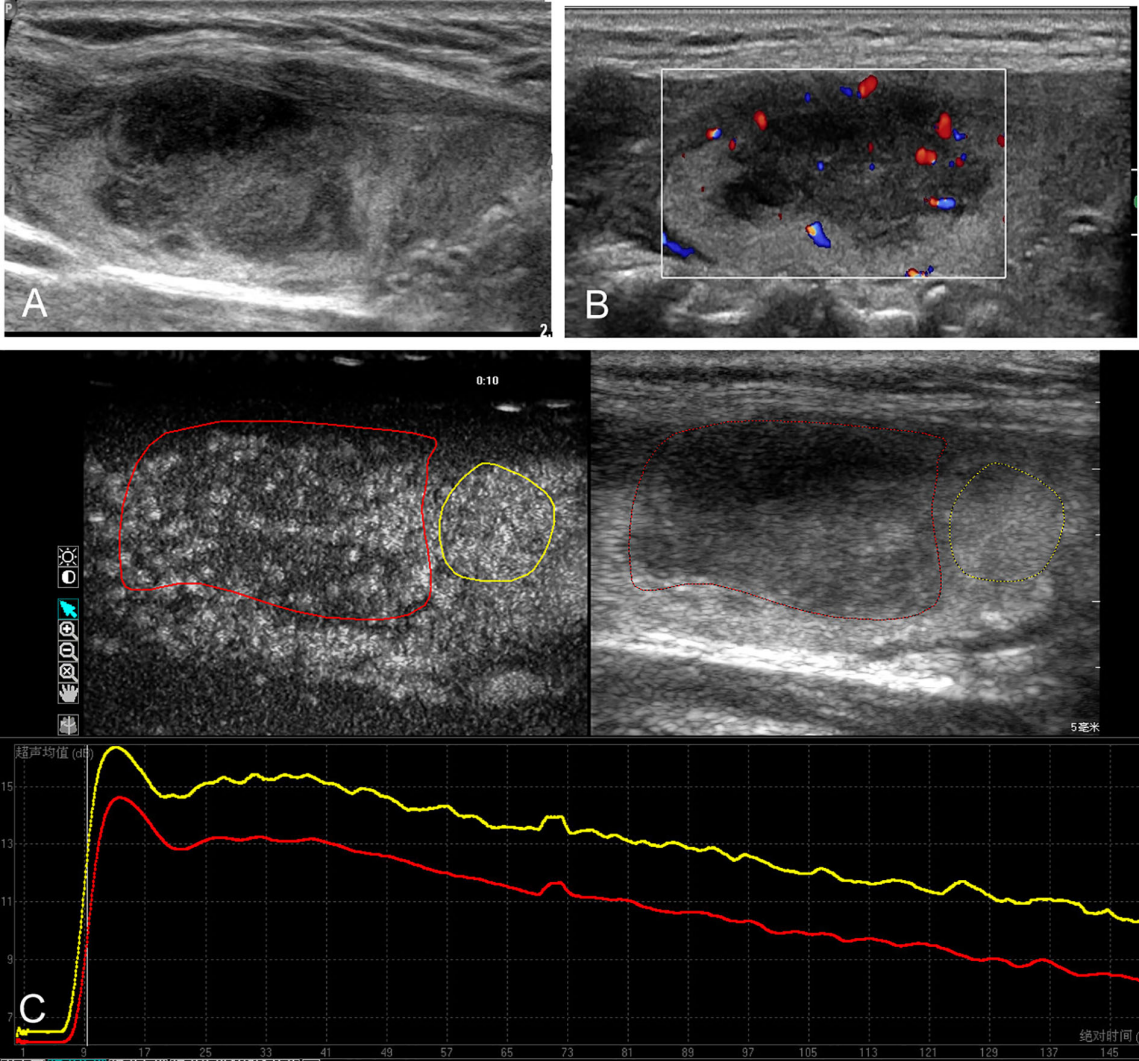
Conventional ultrasound and contrast-enhanced ultrasound images of the primary thyroid lymphoma in the right thyroid lobe of a 67-year-old female with Hashimoto’s thyroiditis background. **(A)** B-mode ultrasound image of a maximum diameter of 28 mm nodule displayed hypoechogenicity with ill-defined margin. **(B)** Color Doppler image revealing the internal and peripheral mixed distributed vascularity of the nodule. **(C)** Contrast-enhanced ultrasound image revealing the heterogeneous hypo-enhancement of the lesion (red circle), with respect to the surrounding parenchyma (yellow circle); time–intensity curves of the lesion (red line) and parenchyma (yellow line) were generated to calculate the corresponding quantitative parameters.

**Figure 2:**
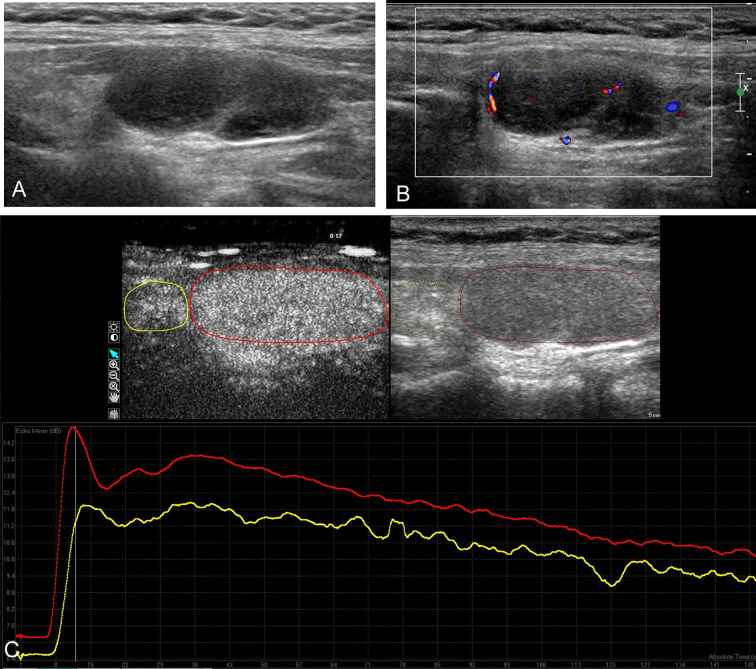
Conventional ultrasound and contrast-enhanced ultrasound images of the nodular Hashimoto’s thyroiditis in the left thyroid lobe of a 26-year-old female with diffuse Hashimoto’s thyroiditis background. **(A)** B-mode ultrasound image of a maximum diameter of 33 mm nodule displayed hypoechogenicity with well-defined margin. **(B)** Color Doppler image revealing the mainly peripheral distributed vascularity of the nodule. **(C)** Contrast-enhanced ultrasound image revealing the homogeneous hyper-enhancement of the lesion (red circle), with respect to the surrounding parenchyma (yellow circle); time–intensity curves of the lesion (red curve) and parenchyma (yellow curve) were generated to calculate the corresponding quantitative parameters.

The CEUS parametric analysis of nodule was performed with Q-LAB software (Philips Healthcare, Bothell, USA), and the time–intensity curve (TIC) within region of interest (ROI) was generated (**Figures 1C** and **2C**). The main CEUS quantification parameters included: peak intensity (PI), defined as the maximum signal intensity; time to peak (TTP), defined as the time it takes to reach PI from the beginning of enhancement; area under the curve (AUC), defined as the area under TIC. Moreover, the ratio indexes of these parameters (PI, TTP, and AUC) were further calculated by the ratio of the ROI of lesion and the ROI of thyroid parenchyma background.

### Histopathological Evaluation

All specimens obtained from biopsy were processed with common histology, and immunohistochemistry. The diagnosis of lymphoma was confirmed according to the World Health Organization classification of tumors of hematopoietic and lymphoid tissues (26). The histopathological results were used as the reference in this study.

### Statistical Analysis

All statistical analyses were performed by SPSS (version 17.0, Chicago, IL, United States) or MedCalc (version 10.4, Mariakerke, Belgium). Continuous variables are presented as mean ± standard deviation (SD), and categorical variables are expressed as the number of cases, with percentage. Independent t-test or Mann–Whitney U test was performed to compare continuous variables between groups. Chi-square test or Fisher’s exact test was used to compare categorical variables as appropriate. Logistic regression was applied, and odds ratios (ORs) were estimated to evaluate valuable US features in diagnosis of PTL with HT background. Receiver operating characteristic (ROC) curves for CEUS parameters and the combination of CEUS parameters and imaging features were created. Areas under the ROC curves (AUROCs) with 95% confidence intervals (CIs) were calculated to assess the capability of CEUS in differentiation of lymphoma and NHT. AUROCs were compared using the method described by Delong et al. (27). The sensitivity, specificity, positive predictive value (PPV), negative predictive value (NPV), and accuracy were calculated according to the optimal cutoff points that maximized the Youden index. Differences were considered significant when P values < 0.05.

## Results

### Clinical and Histopathological Results

In all, 60 patients with 64 nodules were studied. All the included patients consisted of 48 females and 12 males, with an average age of 56 ± 15 (years). The demographic and lab results are summarized in **Table 1**. Among the studied 64 nodules, 31 nodules were histopathologically confirmed as non-Hodgkin’s lymphoma (NHL), including 74.2% (23/31) diffuse large B-cell lymphoma (DLBCL), 19.4% (6/31) mucosa associated lymphoid tissue lymphoma (MALT), 3.2% (1/31) Burkitt’s lymphoma (BL), and 3.2% (1/31) angioimmunoblastic T cell lymphoma (AITCL). The other 33 nodules were confirmed as HT.

**Table 1:** The clinical data of the patients with Hashimoto’s thyroiditis.

Category	PTL (n = 31)	NHT (n = 29)	P value
**Age (y) ^†^**	65 ± 12 (45–87)	48 ± 13 (26–68)	<0.001
**Gender**			0.337
** Male**	8 (25.8%)	4 (13.8%)	
** Female**	23 (74.2%)	25 (86.2%)	
**Thyroid hormones level**			0.553
** Normal**	18 (58.1%)	19 (65.5%)	
** Abnormal**	13 (41.9%)	10 (34.5%)	
**Thyroid peroxidase antibody**			0.866
** Normal**	9 (29.0%)	9 (31.0%)	
** Elevated**	22 (71.0%)	20 (69.0%)	
**Thyroglobulin antibody**			0.655
** Normal**	7 (22.6%)	8 (27.6%)	
** Elevated**	24 (77.4%)	21 (72.4%)	

PTL, primary thyroid lymphoma; NHT, nodular Hashimoto’s thyroiditis.

Unless otherwise indicated, data are numbers of patients, with counted percentage in parentheses.

^†^Data are as mean ± standard deviation, with ranges in parentheses.

### Conventional US Imaging

The main conventional US characteristics are presented in **Table 2**. With respect to the heterogeneous thyroid background, all of the 64 lesions exhibited as hypoechoic solid nodules on B-mode US (**Figures 1A** and **2A**). No calcification was detected in any of them. The mean diameter of PTL was 40.7 ± 14.5 mm (range, 12–64 mm), which was significantly larger than that of NHT with diameter of 24.5 ± 11.1 mm (range, 12–58 mm). In patients with PTL, only one (3.2%) nodule presented as shape of taller than wide. Seventeen (54.8%) nodules exhibited ill-defined margin. Posterior acoustic enhancement and echoic strands were observed in 16 (51.6%) and 12 (38.7%) of the lymphoma lesions, respectively. None of these features on B-mode US showed significant differences with the NHT group. As for the vascular distribution, mixed type of vascularity was more observed in PTL, and peripheral vascularity was inclined to be seen in NHT (p < 0.05) (**Figures 1B** and **2B**).

**Table 2:** Ultrasonographic characteristics of PTL and NHT with Hashimoto’s thyroiditis background.

US manifestations	PTL	NHT	P value
**Size on US (mm)^†^**	40.7 ± 14.5 (12–64)	24.5 ± 11.1 (12–58)	<0.001*
**Nodular number**			0.702
** Solitary**	28 (90.3)	25 (86.2)	
** Multiple**	3 (9.7)	4 (13.8)	
**Ratio of height and width**			1.000
** Taller than wide**	1 (3.2)	1(3.0)	
** Wider than tall**	30 (96.8)	32 (97.0)	
**Margin**			0.209
** Ill-defined**	17 (54.8)	12 (36.4)	
** Well-defined**	14 (45.2)	21 (63.6)	
**Inner echogenicity**			
** Hypoechoic**	31 (100)	33 (100)	
**Posterior acoustic enhancement** ** Present** ** Absent** **Echoic strands** ** Present** ** Absent**	16 (51.6) 15 (48.4) 12 (38.7) 19 (61.3)	11 (33.3) 22 (66.7) 7 (21.2) 26 (78.8)	0.139 0.126
**Calcification**			
** Absent**	31 (100)	33 (100)	
**Vascular distribution**			0.007*
** None**	4 (12.9)	10 (30.3)	
** Internal**	6 (19.4)	1 (3.0)	
** Peripheral**	4 (12.9)	12 (36.4)	
** Mixed**	17 (54.8)	10 (30.3)	
**Enhancement pattern**			0.015*
** Centripetal**	29 (93.5)	23 (69.7)	
** Synchronous**	2 (6.5)	10 (30.3)	
**Enhancement degree**			0.03*
** Hypo-enhancement**	27 (87.1)	21 (63.6)	
** Iso- or hyper-enhancement**	4 (12.9)	12 (36.4)	
**Enhancement homogeneity**			0.001*
** Homogeneous**	1 (3.2)	12 (36.4)	
** Heterogeneous**	30 (96.8)	21 (63.6)	

US, ultrasonography; PTL, primary thyroid lymphoma; NHT, nodular Hashimoto’s thyroiditis.

Unless otherwise indicated, data are numbers of patients, with counted percentage in parentheses.

^†^Data are as mean ± standard deviation, with ranges in parentheses.

*P < 0.05 was considered as a significant difference.

### CEUS Imaging and Quantification Parameters

The CEUS imaging characteristics of lesions are outlined in **Table 2**. Generally, for most of the PTL lesions, contrast agent entered in a centripetal way and presented as hypo-enhancement, as well as heterogeneous (**Figure 1C**). Significant differences were observed between PTL and NHT lesions in the enhancement pattern, degree, and interior homogeneity (all P < 0.05).

The comparison of CEUS quantification parameters and the corresponding ratio indexes are displayed in **Table 3**. In CUES mode, PTL had significant lower values of PI and AUC than those of NHT (both P < 0.05). With respect to the corresponding parenchyma tissue, lymphoma lesions demonstrated lower PI and AUC with ratios of 0.86 ± 0.10 and 0.85 ± 0.12, as well as less time to reach peak intensity with ratio of 0.92 ± 0.08. In NHT group, comparable CEUS parameters of lesions and thyroid parenchyma were observed with ratios of 0.98 ± 0.08, 0.99 ± 0.01, and 0.99 ± 0.09 for PI, TTP, and AUC, respectively. Moreover, the ratio indexes of PI, TTP, and AUC for PTL were significantly lower than those for NHT (all P < 0.05).

**Table 3:** CEUS quantification parameters of PTL and NHT coexistent with Hashimoto’s thyroiditis.

	PI (dB)		TTP (s)		AUC (dB s)	
	Value	Ratio	Value	Ratio	Value	Ratio
**PTL**	12.94 ± 2.53	0.86 ± 0.10	30.55 ± 5.40	0.92 ± 0.08	1646.05 ± 339.56	0.85 ± 0.12
**NHT**	14.86 ± 2.22	0.98 ± 0.08	31.96 ± 6.06	0.99 ± 0.01	1889.40 ± 221.01	0.99 ± 0.09
**P value**	0.001*	<0.001*	0.697	0.001*	0.001*	<0.001*

CEUS, contrast-enhanced ultrasound; PTL, primary thyroid lymphoma; NHT, nodular Hashimoto’s thyroiditis; PI, peak intensity; TTP, time to peak; AUC, area under the time–intensity curve.

Ratio was calculated by the parameter value of lesion vs. corresponding thyroid parenchyma.

*P < 0.05 was considered as a significant difference.

**Figure 3:**
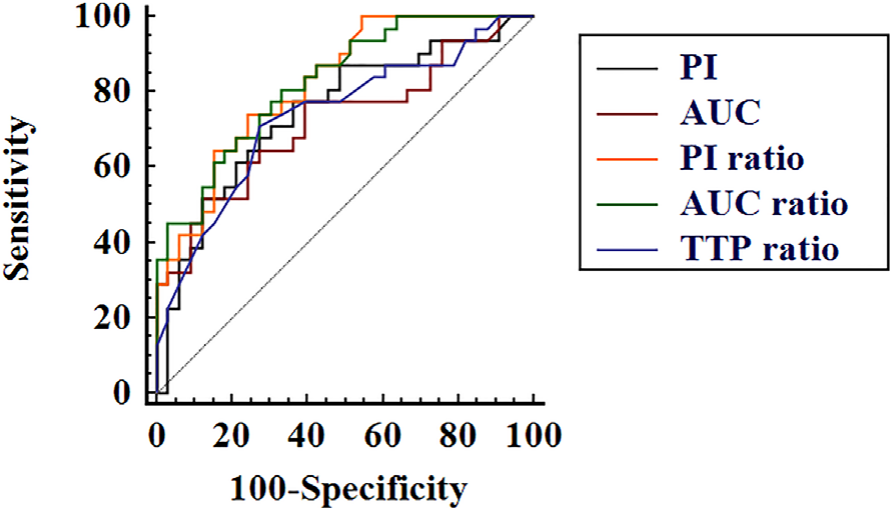
Receiver operating characteristic curves of contrast-enhanced ultrasound quantification parameters peak intensity (PI), area under the time–intensity curve (AUC), PI ratio, AUC ratio, and time to peak (TTP) ratio in differential diagnosis of thyroid lymphoma and nodular Hashimoto’s thyroiditis.

The diagnostic performance of CEUS parameters in distinguishing PTL and NHT with diffuse HT background are illustrated in **Table 4** and **Figure 3**. All the analyzed CEUS parameters showed good capabilities in diagnosis of PTL with AUROCs of 0.72–0.83 and diagnostic accuracies of 70.3–75.0%. No significant differences were observed between independent parameters (P > 0.05 for all).

**Table 4:** Diagnostic performance of CEUS quantification parameters in distinguishing PTL from NHT in patients with Hashimoto’s thyroiditis background.

Parameters	AUROC (95% CI)	Cutoff	Accuracy (%)	Sensitivity (%)	Specificity (%)	PPV (%)	NPV (%)
**PI (dB)**	0.75 (0.62–0.85)	≤14.87	70.3	77.4	63.6	66.7	75.0
**AUC (dB s)**	0.72 (0.60–0.83)	≤1624.85	70.3	51.6	87.9	80.0	65.9
**PI ratio**	0.82 (0.71–0.91)	≤0.93	75.0	74.2	75.8	74.2	75.8
**AUC ratio**	0.83 (0.71–0.91)	≤0.94	73.4	80.7	66.7	69.4	78.6
**TTP ratio**	0.74 (0.61–0.84)	≤0.93	71.8	71.0	72.7	71.0	72.7

CEUS, contrast-enhanced ultrasound; PTL, primary thyroid lymphoma; NHT, nodular Hashimoto’s thyroiditis; AUROC, area under the receiver operating characteristic curve; PPV, positive predictive value; NPV, negative predictive value; PI, peak intensity; AUC, area under the time–intensity curve; TTP, time to peak.

### Valuable Indicators on US Ranking

Indicators of US including conventional US and CEUS were considered valuable when significant differences between PTL and NHT groups were detected. All the valuable indicators were ranked according to their ORs (**Table 5**). Generally, CEUS imaging features and the quantification parameters were more valuable in diagnosis of PTL with higher ORs than that of conventional US.

**Table 5:** Valuable indicators on US and the odds ratios (ORs).

Valuable indicators	ORs	95% confidence interval
**Enhancement homogeneity**	17.14	2.07–142.09
**AUC ratio**	8.33	2.64–26.26
**AUC**	7.73	2.19–27.28
**PI ratio**	7.64	2.51–23.2
**Enhancement pattern**	6.30	1.26–31.66
**PI**	6.00	2.00–18.04
**TTP ratio**	5.60	1.91–16.39
**Enhancement degree**	3.86	1.09–13.70
**Mixed vascularity**	2.79	1.00–7.79
**Size on US**	1.09	1.04–1.14

US, ultrasonography; AUC, area under the time–intensity curve; PI, peak intensity; TTP, time to peak.

### ROC Analysis of Combination Indicators

**Figure 4:**
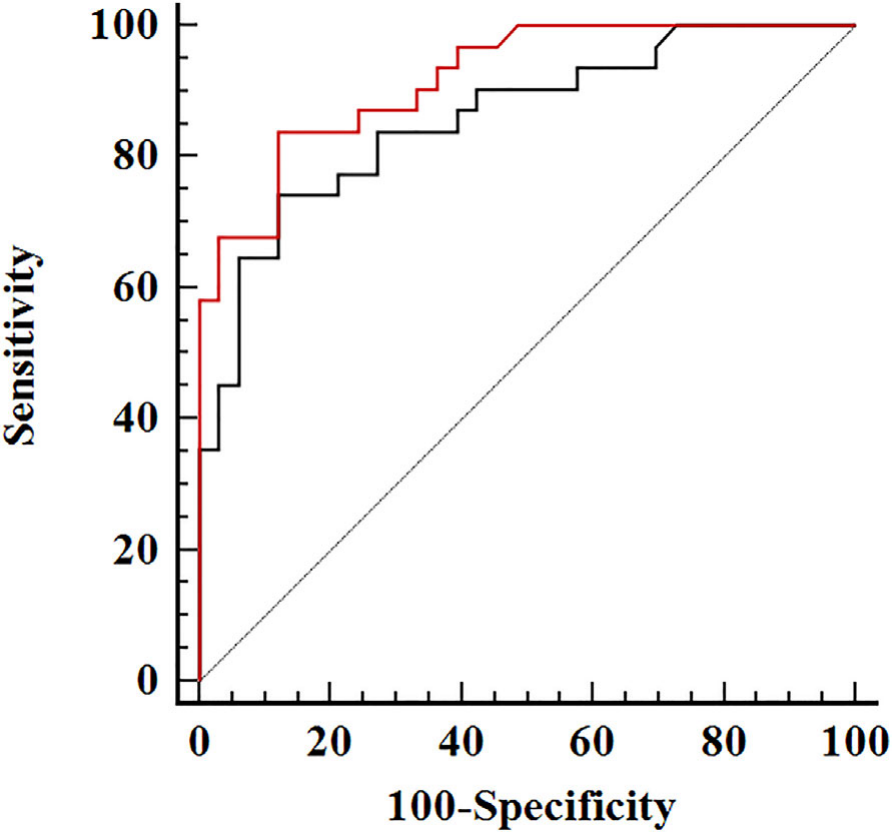
Receiver operating characteristic curves of the combined contrast-enhanced ultrasound (CEUS) indicators in distinguishing primary thyroid lymphoma and nodular Hashimoto’s thyroiditis. The black curve represents the combination of three ratios for peak intensity, area under the time–intensity curve, and time to peak; the red curve represents the combination of the three ratios and the CEUS imaging features including the enhancement pattern and homogeneity.

We further created ROC curves of combined valuable CEUS parameters (ratios of PI, TTP, and AUC), as well as CEUS imaging characteristics (including enhancement pattern and interior homogeneity) (**Table 6** and **Figure 4**). The combination of three ratios of PI, TTP, and AUC demonstrated AUROC of 0.86 (95% CI, 0.75–0.93), which was significantly higher than that of independent indicators of AUC and TTP ratio, with AUROCs of 0.72 (95% CI, 0.60–0.83) and 0.74 (95% CI, 0.61–0.84), respectively (both P < 0.05). Moreover, the combination of CEUS parameter ratios and imaging features showed excellent performance in diagnosis of PTL and achieved the highest AUROC of 0.92 (95% CI, 0.82–0.97), which was significantly higher than that of all single parameters (including PI, AUC, and the ratios of PI, AUC, and TTP) (all P < 0.05). The corresponding diagnostic accuracy, sensitivity, and specificity achieved 85.9, 83.9, and 87.9%, respectively.

**Table 6:** Diagnostic performance of combined CEUS indicators in distinguishing PTL and NHT.

Combination indicators	P value	AUROC (95% CI)	Accuracy (%)	Sensitivity (%)	Specificity (%)	PPV (%)	NPV (%)
**3-ratio combination**	<0.001	0.86 (0.75–0.93)	81.3	74.2	87.9	85.2	78.4
**3-ratio and imaging** **features combination**	<0.001	0.92 (0.82–0.97)	85.9	83.9	87.9	86.7	85.3

CEUS, contrast-enhanced ultrasound; PTL, primary thyroid lymphoma; NHT, nodular Hashimoto’s thyroiditis; AUROC, area under the receiver operating characteristic curve; PPV, positive predictive value; NPV, negative predictive value.

3-ratio combination, indicates the combination of ratios for peak intensity, time to peak and area under time–intensity curve (PI ratio, TTP ratio, and AUC ratio).

Imaging features, includes the enhancement pattern and homogeneity.

## Discussion

Both PTL and NHT are lesions prone to occur accompanied with diffuse HT. Lots of studies have indicated HT as a potential predisposing factor for the development of thyroid lymphoma (3) (4) (22, 28). Matsuzuka et al. (11 *).* once reported a focal HT lesion developed into a typical clinical presentation of malignant lymphoma in a patient with diffuse HT during the 7 years’ clinical follow-up. Another study (29) showed that at least 70% of primary NHL in thyroid was preceded by HT, and at least 53% of them demonstrated findings suggestive of progression from HT through a MALT type, and finally to DLBCL. The chronic inflammation stimulation associated with HT was speculated to act as carcinogens, and the auto-antibody might result in hyperplasia or malignant transformation of lymphoid tissue (3, 30), but the exact mechanisms of how the transformation works are still unclear. The hypothesis that PTL is transformed from HT may be attributable to the extensive overlap in US appearance between lymphoma and NHT.

Ultrasonographic features such as hypoechogenicity, posterior acoustic enhancement, and echoic strands have been reported helpful to predict thyroid lymphoma (10, 13–15, 31). However, these manifestations are not specific, and the results vary among different studies. Ota et al. (10 *).* indicated that the enhancement of posterior on US was the only useful point in diagnosis of PTL, whereas the liner echogenic septa was less likely to be found. In another study (31), heterogeneous hypoechoic with intervening echogenic septa-like structures was reported in 76.9% of the 13 PTL lesions, and the posterior acoustic enhancement was not definite in some cases. In our series, the enhancement of posterior was observed in 51.6% of the PTL cases, which was nonspecific and not significant in differentiation from NHT (P > 0.05). Similar results were also reported by Wang et al. (32 *).*. It has been speculated that the enhanced posterior echo derives from the easy penetration of ultrasound through lymphoma cells which proliferate densely and uniformly. Nevertheless, patients with coexisting diffuse HT usually have fibrotic structures after the destruction of follicular cells by lymphocyte infiltration, which might reflect or absorb ultrasound. Hence, for PTL coexistence with HT, the specificity of an enhanced posterior echo should be further assessed. The linear echogenic strands, which were comparatively less suggested previously in diagnosis of PTL, accounted for 38.7% of the lymphoma lesions in our cases. The discrepancies among studies might be ascribed to the various study populations and the lesions with different sizes. It still remains unclear whether the presence of echogenic strands was associated with the large size of lymphoma (14, 15, 32).

Nodular HT, also known as focal lymphocytic thyroiditis or pseudotumor, has a prevalence of 5% among biopsied thyroid nodules (33). According to the reported surgical pathology archives of Johns Hopkins Hospital for HT cases, 60% (100/167) of the isolated HT (without association with other neoplasms) surgery patients were NHT with suspicious for malignancy (34). On US, the appearance of NHT is variable, and the echogenicity depends on the severity of focal lymphocyte infiltration. In the investigation of 21 NHT lesions by Langer et al. (33), the hyperechoic appearance was more observed in 47.6% of the nodules compared with the hypoechoic ones with percentage of 23.8%, while in several other studies with larger population (22, 23, 35), most of the NHT lesions appeared as hypoechoic with ill-defined margins, which were suspicious signs in diagnosis of malignancy based on the association guideline (36). It should be emphasized that all the enrolled NHT lesions were hypoechoic because of the selection bias of patients whose nodules were suspicious for malignancy and further scheduled for biopsy.

In the current study, no gray-scale US characteristics including shapes, margins, and inner and posterior echogenicity could differentiate PTL and NHT. Similar opinion that lymphoma was indistinguishable from pseudotumor in HT at US was suggested by Takashima et al. as well (22). According to the results, we observed significant larger size of PTL compared with NHT, which was also one of the points that should be taken into consideration in differentiation of these two lesions. However, in clinical practice, small lymphomas were more detected at early phase before the initiation of its rapid growth (10–12). Horii et al. (12 *).* even reported a case of pathologically confirmed early-stage PTL as small as 10 mm. In our study, broad overlap of sizes between PTL and NHT was also found. Moreover, among all the valuable indicators on US in discrimination of PTL and NHT, the size on US ranked the last with an OR of 1.09. Therefore, we could not exclude the diagnosis of PTL based only on the small size of the lesion.

In terms of the vascular distribution of PTL, the studies that can be referred are very limited and the results differ. Wang et al. (32). once demonstrated that a central blood flow pattern would favor the diagnosis of PTL and the increased chaotic vascularity was observed in 61.5% of the 13 PTL lesions by Xia et al. (13), while other scholars considered color Doppler valueless in prediction of PTL (14, 15). In our study in differentiation of PTL and NHT, mixed type of vascularity was more detected in PTL with an OR of 2.79.

CEUS is an effective diagnostic tool for qualitatively and quantitatively detecting microvasculature. Compared with color Doppler technique, CEUS is more sensitive to subtle vessels and has been verified to significantly improve the diagnostic accuracy of thyroid nodules when combined with conventional US. Numerous studies (16–20, 37–39) have demonstrated that heterogeneous/hypo-enhancement indicates malignancy, and homogeneous/ring/iso- to hyper-enhancement suggests benign ones. However, there are few focused on the CEUS features of PTL with coexisting HT. Except for the hypoechoic appearance, PTL has few common features with other malignant nodules on conventional US, such as micro-calcification or taller than wide shape (15). Hence, it is reasonable to doubt whether CEUS is still helpful and the diagnostic criteria for malignant nodules are still applicable in diagnosis of PTL. In our series, based on CEUS imaging, heterogeneous/hypo-enhancement in a centripetal way, which was considered as CEUS features for predicting malignancy, was also valuable in discriminating lymphoma from NHT in HT patients. It should be mentioned that these three CEUS features were also observed in more than 60% of the NHT lesions, which was different from the signs for diagnosis of benignities in thyroid. The manifestation could be explained by the heterogenicity and hypervascularity of the thyroid parenchyma, which might change the enhancement discrepancy between lesions and background.

Generally, in differentiation of PTL and NHT, the parameter ratio of lesion vs. corresponding parenchyma performed better than the parameter of an individual lesion. This is consistent with a previous study (38), in which a ratio model was preferred in identification of thyroid nodules by CEUS. The potential explanation is that the ratio index could eliminate the effect of the thyroid background and individual variations. With the optimal cutoff points of 0.93 for PI ratio and 0.94 for AUC ratio, AUROCs of 0.82 and 0.83 were achieved in distinguishing PTL and NHT. Close cutoff values of PI ratio (0.90) and AUC ratio (0.96) for discriminating benign from malignant solid thyroid nodules were reported by Zhou et al. (38), with corresponding AUROCs of 0.81 and 0.79, respectively. Furthermore, in conjoint analysis of the three ratios and CEUS imaging characteristics, excellent diagnostic efficiency was achieved with AUROC of 0.92, accompanying a sensitivity of 83.9% and specificity of 87.9%. What should be stressed is that the corresponding high NPV of 85.3% indicated the good capability of CEUS in excluding the diagnosis of lymphoma and unessential invasive biopsy in clinic could also be avoided.

There are several limitations in this study. First, for the low prevalence of PTL, the included cases were very limited. Second, all the nodules enrolled might have suspicions of malignancy and were further scheduled for biopsy, which might result in unavoidable selection bias. Third, as the thyroid parenchyma was used as the reference for CEUS analysis, the diffuse type of PTL whose background was not available was not included. Therefore, whether the concluded diagnostic criteria of CEUS are applicable for all types of PTL needs further investigation with larger series.

## Conclusions

In comparison with NHT, PTL was more observed in females with elder age. CEUS performed efficiently in the differential diagnosis of PTL and NHT for patients with coexisting diffuse HT. The heterogeneous hypo-enhancement in a centripetal pattern on CEUS imaging and quantitative CEUS parameters (PI, AUC, and the ratios of PI, AUC, and TTP) were valuable indicators. The conjoint analysis of CEUS imaging features and the ratios of PI, AUC, and TTP improved the diagnostic values.

## Data Availability Statement

The raw data supporting the conclusions of this article will be made available by the authors, without undue reservation.

## Ethics Statement

The studies involving human participants were reviewed and approved by Ethics Committee of West China Hospital. The patients/participants provided their written informed consent to participate in this study.

## Author Contributions

Conception and design of the study: LY, YL, and BM. Ultrasound data acquisition: HZ and YH. Clinical and pathological data collection: XZ and CY. Analysis and interpretation of data: LY, YL, and BM. Drafting the manuscript: LY. Revising and final approval of the version to be published: YL and BM. All authors contributed to the article and approved the submitted version.

## Funding

This research was supported by the National Natural Science Foundation of China (Grant No. 81701702).

## Conflict of Interest

The authors declare that the research was conducted in the absence of any commercial or financial relationships that could be construed as a potential conflict of interest.
